# Medical Items and News of Interest to the Physician

**Published:** 1877-04

**Authors:** 


### For the Use of Physicians.

CULLED FROM THE STANDARD MEDICAL JOURNALS OF THE WORLD.

NOTE.—These formulæ are intended only for the regular practitioner of medicine, and should be employed only by him, or under his immediate supervision.

**Sedative Pills for a Dry Cough.**

Assafœtida, 1 ʒ.

Sulph. morph., 3 gr.

Make thirty pills. Take one or two before going to bed.

**Hæmoptysis Treated by Ergot.**

Dr. J. Williamson, (*London Lancet*, January, 1876), reports fifty consecutive cases of hæmoptysis treated by ergot. Out of these the drug rapidly checked bleeding in forty-four cases. In the other six it failed as did also gallic acid.

**Hoarseness.**

Dr. W. Handsel Griffiths says that a few drops of nitric acid in a glass of sweetened water, a couple of times daily, will be found an excellent remedy for the hoarseness of singers. One of the largest fees ever received by him—so he says—was for this prescription.

**Santonine in Epilepsy.**

An English physician who writes "L. F. P. S. G., L. A. A. Barnsley," after his name, and does it through the courtesy of the editor of the *British Med. Jour.*, reports two patients, aged respectively ten and nineteen, who were relieved of their epileptic attacks by the taking of santonine in doses of one to five grains daily for several weeks.

**Preparation of Ergot for Injection.**

Dr. Wernich points out the disadvantages of the ergot preparations used for subcutaneous injection, and describes a preparation free from these disadvantages.

This preparation is made as follows: Powdered ergot is first deprived of all fatty matter by ether, and then treated with alcohol. A watery extract is then made of the powder so prepared.

Lastly, this extract is further purified by diffusion through parchment paper. It then forms a perfectly clear solution in water, of a brown color. This preparation he proved by experiment to have all the ordinary properties of ergot, and in consequence of its purity is preferable for subcutaneous injection. It keeps well and does not produce pain.—*All. Med. Cent.—Brit. Med. Jour.*

**Priapism.**

Dr. Gibbons, Sr., (*Pac. Med. Jour.*) had derived much more benefit in priapism from veratrum viride than from any other agent He gives the tincture in large doses, at bedtime—seldom less than twenty drops at a dose. His practice is to increase it by a few drops every night till its effect is observable. He has never failed to control priapism by this treatment.

**Large Doses of Ergot.**

At the meeting of the American Gynæcological Society, Dr. Drysdale, of Philadelphia, in speaking of the employment of large doses of ergot in the treatment of uterine fibroids, stated that he had given half-ounce doses of Squibb's fluid extract three times a day for more than a year without producing any injurious effects. When the pain caused by the uterine contractions became too severe, the drug was discontinued until the pain subsided.

**Bronchial Catarrh.**

Dr. Moritz communicated to the St. Petersburg Medical Society the results of his trials of carbolic acid spray in various forms of bronchial catarrh, relating several examples of its utility. Since he had much to do with this spray he found that bronchial catarrh, to which he was formerly much subject, either ceased to appear or was soon cut short. In as small a room as possible he causes half a pound of a 2 per cent. solution of the acid to be sprayed per diem, the night being the time especially to be preferred.—*St. Petersburg Med. Woch.*

**Kava-Kava for Gonorrhœa.**

An infusion of the root of kava-kava (*Piper methysticum*) has long been a popular remedy for blenorrhœa in the Pacific Islands. Three or four scruples, or even more, of the grated root are macerated for five minutes in two pints of water, the whole being frequently shaken up. This water, after filtration, is given in two doses during the day, before or after meals, and repeated every day until a cure is effected. Twenty minutes after the first dose, a pressing desire to urinate is experienced. The quantity of urine is abundant, and it becomes as limpid and as clear almost as water. The pain that was present during the previous micturitions disappears, and a sensation of comfort is experienced in urinating. A cure requires ten or twelve days. The kava, moreover, acts like a bitter tonic. It is pleasant to take, stimulates the appetite, does not derange the digestive functions, and produces neither diarrhœa nor constipation.—*Gazette Medicale de Paris.*

**Incontinence of Urine.**

Mr. Brenchley writes to the *Practitioner* that he has seldom seen much good done in the above disease by belladonna, iron, or bromide of potassium, but has met with much success with the following combination of ergot and iron:

℞

Tinct. ergotæ, 10 drops.
Tinct. ferri perchloridi, 5 drops.
Spts. chloroformi, 5 drops.
Infus. quassiæ., ad. oz. j. ter die sum.

**Jaborandi as a Galactagogue.**

This new medicine has proved to be a powerful sialagogue and diaphoretic, and now a writer in the British *Medical Gazette* announces that he has found it to possess great energy in promoting secretion from the mammary glands, and bringing on a copious supply of milk after other approved means have failed. The dose used was five grains of the powder three times a day, infused in warm water. The medicine is considered, even if it can do no good, to do no harm to either the infant or its mother.

**Lotions for Urticaria.**

Prof. Hardy recommends (*Union Med.* May 20), the following lotion, to be applied several times a day in order to allay the itching in urticaria: Chloroform ten, and oil of sweet almonds thirty parts. In obstinate cases he prescribes corrosive sublimate, one-tenth to one-seventh of a part; alcohol, ten parts, and distilled water, ninety parts. He gives also internally alkaline medicines, and if these do not prove efficacious, he resorts to arsenic.—*Med. Times and Gaz.*

**Physiological Action of Jaborandi.**

Dr. Felippo Cesari, of Rome, in the *Gazetta Medica*, of that city, has an article entitled "Physiological Experiments and Clinical Notes on the Polycarpus Pinnatus or so-called Jaborandi," in which he comes to the following conclusions: 1. That the jaborandi of Coutinho has an elective action on the salivary glands. 2. That it has a secondary action, but less constant and copious, upon the sudoriparous glands. 3. That it has well-marked diuretic action. 4. That it produces no effect whatever on the circulation, nor on the generation of heat, nor (except in an unimportant degree), on the respiration. 5. That its action is most efficacious when it is injected into the veins. 6. That its after effects are distinct debility, with an imperious thirst, directly proportioned to the action of the emunctories. These effects, however, are transitory, and soon disappear under restorative diet.

**Treating Blisters by Osmosis.**

M. Ungerer recently saw an extensive scald, which had for twelve hours been treated with cold water without relief from the agonizing pain, or reduction of the swelling. The experiment of immersing the limb in a saturated solution of salt was followed by most surprising relief. The abatement of the pain was immediate, and in four hours both the pain and swelling were gone. The next day the hand differed from the other only by a very slight swelling and redness.

**Fetid Feet.**

A very obstinate case of this complaint, in a workman, is reported in the *Bull. de Ther.* by Dr. Ortega. In the manufactory in which he worked he was avoided by his fellow-workmen, and when he entered a room the window would be opened. He had consulted several physicians but without success. The epidermis of the sole of the foot was white and macerated, and there were little ulcerations at the clefts of the toes and around the nails. M. Ortega advised him to apply compresses soaked with a solution of chloral, which had the effect of rapidly destroying the smell and curing the ulcerations.

**Prickly Heat.**

Persons subject to this annoying affection will be glad to learn that Surgeon-Major Dr. J. G. French, of the Indian medical service, in a contribution to the *Indian Medical Gazette*, says that we can cure prickly heat in three or four days by the application of a solution of sulphate of copper. This should be of the strength of about ten grains to the ounce of water, and the solution should be applied daily, or oftener, by means of a camel-hair brush or bit of sponge tied on the end of a stick. It is best applied after the morning bath, when the skin has been well rubbed with the towel, and it must be allowed to dry on the skin before dressing. Dr. French states that he has used this application for over thirteen years, and, when regularly and properly applied, he has never known it to fail.

[It will be a comfort to persons who are yearly doomed to endure for about four months each summer the frightful torments of this apparently incurable affection, to learn that there is balm in Gilead. When the body and limbs of a man look in color like a boiled lobster, his constant longing and hope is that cooler weather will come and relieve him of his distress. The foregoing may be good, but what a piece of work to wash from head to foot daily, with a camel's hair brush, in a solution of blue vitrol!—ED.]

**For Chilblains.**

I. Balsam of Peru, ʒ iss.

Alcohol, ℥ iss.

Dissolve and add

Hydrochloric acid, ʒ ss.

Compound tincture of benzoin, ℥ j.

II. Chloride of ammonium, ℥ ss.
Water, ℥ iv.
Hydrochloric acid, f. ʒ j.
Alcohol, f. ℥ iss.
Mix. Apply morning and evening.

**Carbolized Bran in Compound Fractures.**

Dr. L. Mason, of Brooklyn, states: "The advantages possessed by bran properly mixed with carbolic acid as a dressing in compound fractures are these: 1. The discharge is disinfected as it flows into the bran. 2. We have a dressing that is 'germ proof,' and one that notably limits suppuration. 3. We secure the anæsthetic properties of the acid. The method of carbolizing the bran is very easy, simply adding crude carbolic acid to the quantity of bran to be carbolized, stirring it at the same time. A little experience will decide how much of the acid a given quantity of bran will require; an excess should be avoided. The bran retains the properties of the acid for some time."—*N. Y. Jour. of Med.*

**Camphorated Ether in Erysipelas.**

Dr. Cavazzani gives the following formula in the *Gazzetta Med. Italiana Provincie Venete:*

℞
Camphor, 15 grs.
Tannin, 15 grs.
Ether, 2 ʒ.

This is painted every three hours, and sometimes oftener over the affected parts. The author says he has never seen this method fail, even in the most severe cases, in which ataxic and adynamic symptoms had already appeared. The fever soon diminishes, and the local erysipelatous process is arrested in two or three days.

In some cases of phlegmonous erysipelas, which Dr. Cavazzani had under his care, this treatment arrested the progress of the disease. Trousseau prescribed this drug only in cases of circumumbilical erysipelas in new-born children, and Guibout did not use this solution in phlegmonous erysipelas, or in that affecting the face, fearing in the latter case that the meninges would become affected. In seeking an explanation of the action of the remedy, Dr. Cavazzani supposes that erysipelas is nothing else than a lymphatitis, and that the tannin exercises an astringent action on the cutaneous capillaries.

**Phosphorus in Leucocythæmia and Allied Diseases.**

In view of the extremely intractable nature of these affections, it may be of interest to direct the attention of our medical readers to the discussion which recently occurred in the Clinical Society of London, on the effect of phosphorus upon them.

Although the number of cases cited by the various speakers was not large, the success which attended the use of the remedy in a few of them warrants its trial. The report of the discussion may be found in the *British Medical Journal.*

**Syrup of Liquorice Root.**

A. P. Brown, *Am. J. Phar.*

Liquorice root, 4 troy ounces.
Cold water, q. s.
Water of ammonia, 1 fluid ounce.
Granulated sugar, 13 troy ounces.

Grind the root in a mill and place it in a wide mouth bottle with tightly fitting stopper, pour upon it one pint of water, mixed with the water of ammonia, macerate for 48 hours, then transfer it to a funnel, allow the liquid to drain from it, and add sufficient water until two pints of liquid have passed; allow it to stand until the particles have subsided, then decant and evaporate to 8 fl. oz., filter, and having added the sugar, dissolve it with the aid of heat.

**The Caustic Properties of Bromide of Potassium.**

Mr. Peyrand has tried the escharotic properties of bromide of potassium upon malignant and other growths, either by means of injections into the tumor, or by the application of the powdered salt to the raw surface. The action of the salt is completely resisted by the tegument. His first clinical experiment on the subject took place in April, 1874, when, by means of daily applications of powdered bromide, he effected the removal, within twenty-eight days of an epitheliomatious growth on the face. He has since had equally good results from this treatment of atonic ulcers of the legs, rapid cicatrization following the separation of sloughs produced by the application. In such cases he uses either the powder or an ointment of one part in five, or a mixture (one in ten) of glycerine and the bromide. In many skin affections, as chronic eczema, pityriasis and acne, in phagedæna, ulcerative stomatitis, and many other local inflammatory disorders, he has found it of use. As a local hæmostatic, a solution of one in fifty has served for epistaxis; and as a general hæmostatic, its success in many cases of hæmoptysis and metrorrhagia was very marked, where ergot, perchloride of iron, and rhatany had failed.

**Internal Administration of Chloroform.**

M. Jaillard, a French army *pharmacien*, after alluding to the difficulties that occur in the internal administration of chloroform, states that the following simple procedure is by far the best: The prescribed quantity of chloroform should be poured into from 100 to 120 grammes of milk (which may be either pure or sweetened and aromatized with a few drops of cherry laurel water), and then briskly stirred. The chloroform in this way becomes easily divided into an infinity of minute globules, exactly resembling in appearance the fat globules which exist in the milk, in the midst of which they remain suspended for an indefinite period. *Rècueil de Med. Militaire.*

---

**Salicylate of Soda in Gout and Neuralgia.**

The statements that have recently been made by several writers, that salicylic acid and salicylate of soda, when given in acute rheumatism, relieve the pain more certainly than the swelling, indicate the trial of these substances in affections where pain may be a chief characteristic. Dr. C. Cunzi (*Deutsche Zeitschr fur prakt. Med.*) recommends salicylate of soda as a means of rapidly relieving the pain of gout. In two cases of gout of the foot a single dose of one drachm was followed, in three hours, by complete cessation of pain; the swelling, however, remained ten days longer. Dr. L. Hoffmann (*Berliner Klin. Woch.*) has found it remarkably efficacious in gout of the hands and feet, and relates successful cases of its use in sciatica, tic douloureux, and intercostal neuralgia. He recommends half a gramme to be taken in a gelatine capsule every hour.—*Br. Med. Jour.*

---

**Tuberculosis Treated by Inhalations of Nitrogen.**

The *Doctor* states that Dr. Steinbruck declares in the first stages the disease is cured with positive certainty by systematic inhalations of nitrogen, if used long enough. He does not know whether relapses occur. In the second stage improvement and cures are attained to an extent hitherto impossible. The younger the patient the better is the withdrawal of oxygen borne, and the more certain is the result. In the third stage the inhalations are dangerous. All beneficial treatment of tuberculosis must depend upon quieting of the nervous system. The inhalations are practiced in an air tight cabinet, under the supervision of the physician, and the effects looked for are easier and deeper breathing, with coughing up at first of mucopurulent matter. Subsequently, the cough should stop, the pulse and temperateture fall, and the nervous system be calmed.

**Boudet's Depilatory.**

Crystallized hydrosulphate of soda, 3 drachms.
Powdered quick lime, 10 drachms.
Starch, 11 drachms.

Mix and keep in a well stoppered vial. To apply it, make the powder into a paste with a little water, and spread it with a wooden or bone spatula on the part to be deprived of hair. After two to four minutes, wash it off with water. The length of time the paste is to be left on varies according to the coarseness of the superfluous hair.

---

**Dr. Ferrier's Remedy for Cold in the Head.**

We have so few things that can be of much service in arresting or relieving this disagreeable and common complaint, that we print again Dr. Ferrier's formula for a snuff, which is reported to work very well:

℞

Bismuthi trisnitratis, ʒ vi.

Pulv. acaciæ, ʒ ij.

Morphiæ muriatis, gr. ij. M.

To be used occasionally as a snuff.

---

**Cream of Camphor.**

Ch. Griffith, *Am. J. Phar.*

Castile Soap, ℥ iss.
Water of ammonia, fl. ℥ iss.
Camphor, ʒ vi.
Oil of Turpentine, fl. ʒ vi.
Chloride of ammonium, ℥ iss.
Water, fl. ℥ xij.

Dissolve the soap-shavings in one-half of the water previously mixed with the ammonia, and the ammonium chloride in the other half; mix the solutions well and add the camphor dissolved in the turpentine; then agitate briskly until the liquids are united and form a perfect emulsion. Other active medicines may be added when indicated.

---

**Hydrobromic Acid Cough Mixture.**

Dr. J. Milner Fothergill says the following is a really charming cough mixture, efficient as well as palatable:

℞

Sp. chloroformi, ♏ xx.

Acid. hydrobromic fl. ʒ ss.

Syr. scillæ, fl. ʒ i.

Aquæ, ad. fl. ℥ i.

Ter in die. Any other acid is, he says, very agreeable; but the hydrobromic acid, from the effect of the bromine upon reflex mechanism, allays the cough, often so troublesome. It possesses much the same action as opium, without the ill effects upon the digestive organs or the bronchial secretion.—*Medical Times.*

**Typhoid Fever.**

Dr. Duboue, of Pau, (France), has recently related his experience in the treatment of typhoid fever by ergot. What next will this universal specific be credited with curing? He gave it to eight men and seven women. There were two deaths—the others recovered, though some were admitted into the hospital in the last stage of the affection. Now we should like to know if the ergot contributed any impulse towards a cure? If it did, then on what physiological principle? Typhoid fever is classed as a zymotic disease; the blood is contaminated with a special viris, and ulceration of the bowels is its constant and almost pathognomonic symptom. How would ergot meet such a case?

---

**Disinfectant.**

Dr. H. Mallory, of Hamilton, O., recommends the following as a disinfectant:—For twelve years I have used extract of logwood as a disinfectant and deodorizer in cancer. I use it in the following manner: Powdered logwood and hog's lard, of each, two ounces. To be mixed and made into a pomade, spread on lint and applied to the sloughing ulcer; the effect is magical, all the odor will disappear in half an hour. The astringency of the logwood will suppress the discharge. No other known agent will fill the indications so well, and yet I have not found a single member of the profession who had any knowledge of the agent until I suggested it. Will some of your numerous readers give it a trial and report the results.

---

**Salicylic Acid in Acute Rheumatism.**

At the December meeting of the Medical Society of the Royal College of Physicians in Ireland, Dr. J. W. Moore communicated the results of his experience of this remedy in the treatment of acute rheumatism in the Meath Hospital and County Dublin Infirmary. Detailed observations of six cases were given, and brief notes of ten cases treated by other methods were appended for the sake of comparison. The ten patients spent an average period of 26.6 days under treatment in hospital. The average duration of the stay in the wards of those who were given salicylic acid was 14.8, and this, too, although most of the patients treated by it were purposely kept under observation for many days after the symptons had disappeared. The mean duration of the sojourn in the hospital is now only about *one-half* what it used to be. Again, an examination of the clinical charts in the cases shows that under the ordinary methods of treatment the average number of "days of pyrexia," or days on which the axillary temperature reached or exceeded 99° Fahr., was 19.3. Under salicylic acid, on the contrary, it was 5.5 days. So that the symptoms of feverishness was but *one-fourth* as persistent in the second series of cases as it had been in the first. If it were fair to base any definite opinion on so few observations, the author would give the following conclusions as to value of the new treatment: 1. Salicylic acid appears to be a valuable and almost specific remedy in the treatment of acute rheumatism. 2. After the administration of a few moderate doses of five grains each, given at hourly intervals, a marked amelioration of the symptoms usually occurs. Thus, the temperature and pulse begin to fall, the swelling and pain of the affected joints subside, and the patient sleeps. 3. The above doses—*i. e.*, of five grains each—are quite sufficient to produce an impression on the disease, while the patients make but little complaint either of the frequency of the dose or the taste of the medicine. 4. When pushed far, it sometimes causes singing in the ears and diaphoresis. Under these circumstances its administration should be temporarily suspended. 5. To prevent relapse, it should be given for some days, but at gradually lengthening intervals. 6. Finally, as to its probable action as a preventive of the dangerous cardiac lesions of acute rheumatism, the author could only endorse the words of Dr. Coates, of Belfast, in a recent paper: "I think it can hardly be denied that medicines which cut short the disease, as I believe there can be no doubt salicylic acid does, must render the liability to these complications less."

---

**Acne.**

M. Rodet, of Lyons, prescribes the following treatment in acne. Friction is to be made every evening over the acne papules with the following:

℞ Adipis, ʒ v.

Sulphuris,

Acid. Tannici, āā gr. viij. ad xv. M.

In the morning the face is to be bathed with warm water, to which a little bay rum has been added, the proportion being increased from day to day until until it amounts to one-third. M. Doyen, of Lyons, recommends bathing with the following:

℞ Hydrarg. bichloridi, gr. xxx.

Tinct. lavandulæ, f. ʒ ijss.

Aquæ distillatæ, f. ℥ x. M.

M. Hardy uses this formula:

℞ Potassii sulphureti,

Tinct. benzoini, āā ʒ ijss.

Aquæ, f. ʒ x. M.

Two teaspoonfuls in a glass of warm water to be used externally.—*Med. Times, from La France Med.*

**Action of Chloral on the Rectum.**

It would appear that chloral is one of those agents which act with nearly as much energy when introduced in the rectum as when taken into the stomach. In a case of puerperal convulsions, to which we had been called in consultation, a solution of bromide of potassium with hydrate of chloral, which could not be swallowed by the patient, was injected into the rectum, with the effect of allaying spasm promptly and decidedly. It was repeated in the same case with excellent results. Since that time other trials of chloral as an enema have confirmed its value in this mode of administration. The quantity of thirty grains in two or three ounces of water will generally be sufficient for a single injection.—*Pacific Med. Jour.*

---

**Asthma.**

Dr. W. B. Furman says, in the *American Medical Weekly*:—

The following remedy I can recommend as sure to quiet any paroxysm of asthma for a time, and in uncomplicated cases I am seldom called on to give more than one or two doses:—

℞ Sulph. atropiæ, gr. j.
Aquæ bullientis, ℨ vij. M.
Dissolve and add
Sulph. morphiæ, gr. xv. M.

Sig.—Use from six to ten drops, hypodermically.

This preparation may, with the utmost safety, be used during the most severe paroxysms, and never fails to gratify the suffering victim and satisfy the physician.

The above solution will keep indefinitely with the addition of two drops of carbolic acid.

---

**Eucalyptus Globulus in Dropsies.**

Dr. J. B. Leary, in the *Proceedings of the Medical Society of King's County*, for September, says that nearly four years ago he prescribed eucalyptus as a specific in gonorrhœa. Then he noticed also, its remarkable diuretic effect. He now reports the more remarkably successful use of ten-minim doses of the fluid extract in four cases of dropsy of long standing—the dropsy in one case being due to Bright's disease; in the second to cardiac hypertrophy by dilatation; in the third to cardiac disease; and in the fourth to cardiac hypertrophy. The doses were never increased beyond ten minims three or four times a day, but in some cases diminished to eight minims—the system at no time tolerating it.

The doctor adds that he has also given the medicine in a great many cases of passive congestion of the kidneys, and always with benefit. Patients while taking the drug would sometimes complain of a very severe congestive headache, accompanied with tinitus aurium. The appetite was improved and in some cases a laxative condition of the bowels was produced.

It should be remembered in prescribing it that the drug contains a resin which is precipitated by many other agents. Dr. Leary's most frequent combination was with digitalis.—*Virginia Medical Monthly.*

---

**Beach-tar Creasote in the Treatment of the Expectoration of Phthisis.**

Dr. Georges Daremberg has first made a chemical analysis of the sputa of phthisical patients with great care; he has shown that these sputa may contain almost as large a quantity of phosphates and chlorides as the urine, and that expectoration in these cases is one of the ways by which the products of denutrition are expelled; this expectoration, however, is not only one of the ways, but also one of the causes of this denutrition, which is indicated by precise prognostic and theraputic data. Beech-tar creasote has been employed in France by M. Bouchard, and in five cases of advanced phthisis the results have been favorable; the expectoration has quickly stopped. From 20 to 40 centigrammes (about 3 to 6 grains), of creosote per diem were given. According to Hlasewetz and Barth, this beech-tar creasote is a combination of creosote ($C_8H_{19}O_2$) with a carburetted hydrogen.

---

**Diptheria.**

Dr. W. E. Anthony, of Providence, R. I., writes to the *Medical and Surgical Reporter*, as follows:

While I do not consider the sulpho-carbolate a specific in this disease, I do think that its judicious and persistent use will, in many cases, be followed by an amelioration of its symptoms.

Just what its mode of action is I am not fully prepared to say. It is possible that it acts as an antidote and eliminative to the peculiar blood poison which is the cause of the disease. It is a stable salt, parting with its acid only when brought in contact with the fluids of the body. In one case, where a large quantity had been used for several days, the odor of carbolic acid was plainly perceptible in the urine. The remedy may be used in every form and stage of the disease, in doses of from one to ten grains, repeated every one, two, three or four hours, according to the necessities of the case. The proportion of acid in the salt is about one-fourth, which will determine the dose.

I have given as high as one hundred and twenty grains in twenty-four hours, to a child seven years old. It may be combined with quinia sulph., tinct. ferri mur., ammonia carb., or given in brandy, whisky, wine, syrup or any aromatic water.

A very good way to dispense it to children is to mix it with sugar and let them eat it. For adults I sometimes use the "cachet de pain." My rule is to begin the administration of the remedy as soon as the disease is recognized, and to continue it in increasing doses until its effects upon the disease is manifest, then gradually diminish the dose and increase the intervals between the doses.

In addition to the use of the sulpho-carbolate, I always use tonics and stimulants freely, and nourishment, in a concentrated form, such as beef extract, cream, etc.

The local treatment is directed to the removal of the false membrane and the reduction of the local inflammation. This result is obtained, first, by hastening the natural progress of exfoliation; second, by the use of such remedies as will destroy the micrococci and dissolve the pseudo-membrane.

---

**Phthisical Laryngitis.**

Dr. J. Sawyer says, in the *British Medical Journal:*

Phthisical laryngitis is a very painful malady; and, when it has passed into its second stage, it is always fatal. It is difficult to give much relief by treatment. But I wish to speak very confidently of the good results which arise from the frequent application to the larynx of a solution of nitrate of silver; one drachm of the salt to an ounce of water. In the first stage, this remedy stimulates the nutrition of the larynx, and so combats the local anæmia; in the second stage it reduces the tumefaction; in the third it checks the ulceration. In all, it deadens the morbid and painful sensibilities of the affected parts. Dysphagia always arises in this disease. It is often a very serious symptom. The tumid and tender larynx makes deglutition difficult. This condition is promptly relieved by the local application I have recommended.

---

**Tubercular Consumption.**

The hypodermic syringe is becoming a more common instrument in the hands of medical men than the lancet was forty years ago. Here is the last surprising exploit in subcutaneous injection. Dr. Schmitzler, of Vienna, states in the *Medical Press* that he has used carbolic acid in more than one hundred cases. The strength employed in water was one to fifty, or sometimes one to one hundred daily, for several weeks. There is nothing said of recovery, nor whether it hastened death. Of course, it naturally acted as a strong stimulant of the heart and arteries. But what else would it be likely to do? Could it probably arrest the formation of tuberculous matter in the blood, or wherever this substance may be produced? We doubt it. In phthisis there is a blood poison—a real entity in all probability—but pathologists know as yet too little about it to determine whether it is composed of living germs or not. If it is, then carbolic acid might possibly destroy it, or at least some one of the other antiferments might do so that have recently been so much discussed. We shall wait for more light. It cannot yet be admitted that tubercular consumption can be cured by such means.

---

**Aspiratory Puncture of the Bladder.**

In a communication to the Hufeland Medical Society, Dr. Furstenheim called the attention of his colleagues to the great value of Dieulafoy's instrument for capillary puncture of the bladder, and especially in localities where practitioners may not have had practice in passing catheters in difficult cases. Here is a means of emptying the distended bladder, easy of employment, unattended with danger, and infinitely to be preferred to the ordinary hypogastric puncture of the organ, which, in discussions in recent times, has been spoken of far too lightly. So little danger is there in passing Dieulafoy's capillary trocar through the integuments into the bladder, that Lucke recommends it in the diagnosis of stone in the bladder when the ordinary means fail; and Socin does not hesitate to employ it in the same case several times daily. Deneffe and Van Wetter have collected fifty-seven cases in which it has been employed without any fatal issue; but Dr. Furstenheim is not aware that this small yet important operation had ever been performed in Berlin, and he therefore brought before the Society a remarkable instance of its great efficacy.—*Berlin Klin. Woch.*

---

**Secret Pile Remedy.**

Professor Edmund Andrews, of Chicago, says, in the *Journal and Examiner:*—A number of men have been itinerating in Illinois and the adjacent states, treating hemorrhoids by a new method. The secret has been sold to various physicians and other persons, at prices varying from fifty to twelve hundred dollars, and some of the purchasers have left a good practice in the expectation of making a fortune by traveling about and applying the remedy.

Investigation has placed the plan more fully in my possession, and I give it here, for the benefit of all concerned.

The first thing is to have a good hypodermic syringe, kept in perfect order, with sharp, delicate pipes. The fluid used is strong carbolic acid dissolved in any bland fixed oil. The proportions are usually as follows:—

℞ Crystallized carbolic acid, ℥ iij.
Pure oil, f. ℥ j. M.

Some of the itinerants use equal parts of the two ingredients, and some of them substitute glycerine instead of oil, and at least one of them has tried a preparation of ergot.

When the piles are internal, and not readily brought down, a Sims' speculum is employed to uncover them. The operator generally takes only one pile at a time, always selecting the uppermost first, and injects into its interior from four to six drops of the carbolized oil, or rather the oleized carbolic acid. The injection turns the pile white, probably coagulates the blood in its vessels, and results in its shrinking away without the inflammation being severe enough at any one time, as a general thing, to prevent the patient from attending to his business. The well known power of carbolic acid to act as a local anæsthetic, antiphlogistic and anti-suppurative, favors the progress. When the irritation of the first injection has measurably subsided, another pile is attacked in the same way, and, as the patient cannot see the syringe, he supposes that he has not been subject to any "operation," which is a great satisfaction to him. The itinerants often call their plan "painless," but it proves to some persons atrociously distressing. The result is in many other cases excellent, so that the plan may turn out to be worthy of a permanent place in the treatment of hemorrhoids.

---

**Malarial Chill.**

Dr. Benjamin Lee writes to the *Med. and Surgical Reporter*, of Philadelphia, a note on using chloral hydrate in ague, which looks very much like stealing a march on disease by practical deception of the patient. We suppose, however, that physiologists will be able to find a satisfactory scientific reason that may show that the action of the remedy was a good deal more than bringing the patient to a state of unconsciousness. A young lady, writes Dr. Lee, was suffering from high fever, with delirium and intense headache. As I was already attending two cases of herpes gutturalis in the same family, I concluded that she had caught the prevailing epidemic; and, although she would confess to no sore throat, put her on full doses of chlorate of potash, dissolved in water acidulated with hydrochloric acid. The following morning she was free from fever, but not from headache; was excessively weak; had slept little during the night, but had perspired considerably.

Before the fever came on, on the day previous, she had suddenly, while at the piano, been seized with a severe chill, which had continued for an hour. I prescribed a mild saline cathartic, and next morning ten grains of quinine in solution at 6 A. M., and fifteen more at 10 A. M. The chill, however, came on with even greater violence than the first; as described to me, it must have been almost pernicious in its character. When I saw her late in the afternoon, she was again delirious; the heat of skin was excessive; the pulse could with difficulty be counted, and she complained piteously of her head.

In conversation with a highly intelligent clergyman, who had spent many years in Texas, he alluded to the plan adopted by physicians there, in such cases, of giving full doses of opium in anticipation of the chill, and causing the patient to sleep through the time in which it should occur. I at once determined to give the method a trial, substituting chloral for opium, as being essentially anti-spasmodic in its action. Giving quinine as before, about fifteen minutes before the time at which algid symptoms had begun to declare themselves previously, I caused her room to be darkened, and all unnecessary noises about the house to be stopped; administered twenty grains of chloral hydrate, dissolved in camphor water, and left her. In ten minutes I returned to her room, with the intention of repeating the dose if there was any sign of excitement about her. She was already, however, sleeping quietly, with pulse and skin natural. I visited her at intervals of about an hour, always to find her in the same placid sleep, from which she awoke, much refreshed, about 4 P. M., the chloral having been administered soon after 10 A. M. There was no return of the chills for that season.

Seven months later, on the 10th day of May of the present year, she was again seized with a severe chill. Profiting by my experience on the former occasion, I now directed that she should be closely watched on the day of the recurrence, the quinine administered as before, and at the first indication of the invasion (which previously had not been awaited), the usual dose of chloral administered. The person to whom I entrusted the case had been with the patient in all the previous attacks, and was an intelligent and trustworthy observer.

